# Application of Blockchain Technology in Production Scheduling and Management of Human Resources Competencies

**DOI:** 10.3390/s22082844

**Published:** 2022-04-07

**Authors:** Barbara Balon, Krzysztof Kalinowski, Iwona Paprocka

**Affiliations:** Department of Engineering Processes Automation and Integrated Manufacturing Systems, Faculty of Mechanical Engineering, Silesian University of Technology, Konarskiego 18A Str., 44-100 Gliwice, Poland; barbara.balon@polsl.pl (B.B.); krzysztof.kalinowski@polsl.pl (K.K.)

**Keywords:** blockchain technology, scheduling, management, human resources, competencies

## Abstract

Today, enterprises are multitasking, with branches set up all over the world. Virtual enterprises are created to make better use of existing resources, improve the quality of manufactured products and agilely respond to customer requirements. In order to fully meet the requirements of enterprises, a decentralized structure of data registration and transmission and authentication of network users is needed. The information collected via the Internet of Things and flowing based on the properties of the Blockchain (BC) network facilitates enterprise resource planning and enables the integration of internal processes, especially when planning, changing the current or introducing new production. The aim of this paper is to present the concept of using a common data register in BC technology, which enables a number of applications related to the automation of the process of selecting human resources for production tasks. The paper presents an analysis of the problem related to the integration of production scheduling and human resource management with blockchain technology. Also presented is a literature analysis on scheduling, blockchain technology and data storage in the blockchain network. The analysis presents how the blockchain network works and how exactly it fits into production engineering with its advantages and disadvantages. An employee evaluation method based on the resource work history and determination of its current value within individual competencies is presented. The integration of production scheduling and human resource management with the use of BC technology is simulated. The most important advantage is faster and more effective planning thanks to the elimination of all intermediary channels in the flow of production transactions. Production tasks are balanced with production capacity in entities belonging to the virtual enterprise in parallel. For future research, different online planning algorithms will be developed and compared to achieve consortium members’ consensus on production and human resources planning.

## 1. Introduction

Globalization affects an enterprise, regardless of the country in which it operates, its economic, political or social situation. The process of globalization has started an era of innovation, which is mainly characterized by strong market competition, shorter product life cycles and a great variety of products [1]. Modern technologies also contribute to the multitasking of enterprises which, without the slightest fear of stagnation in access to their own information, set up their branches all over the world. Business intelligence tools and enterprise management systems (ERP—enterprise resource planning) are often used to integrate and share internal data, which, despite their popularity, must be expanded with additional systems. In order to fully meet the requirements of enterprises, the APS (Advanced Planning and Scheduling) system is a good support solution [2,3,4,5].

The identity of a modern company is primarily shaped by the applied innovative technologies, such as blockchain or the Internet of Things. Currently, corporate servers are one of the most popular methods of storing production data. An equally popular method of data storage is the circulation of information in the so-called “Clouds” that allow one to store data in cyberspace outside the enterprise. Cloud space, compared to traditional data storage on servers, seems to be more advantageous mainly due to the fact that it eliminates: financial resources related to fees for servers, electricity bills, the need to find additional rooms serving as server rooms, fees for IT services related to the operation of corporate networks, fees for services related to cybersecurity and the possession of current utility licenses.

Nakamoto [6] defines blockchain as a distributed ledger that contains transactions confirmed by cryptographic digital signatures and is grouped into blocks. The main advantages of BC are:

- Decentralization—in blockchain, each transaction is confirmed by a decentralized network based on consensus algorithms, therefore there is no need for a central supervisor that maintains data consistency;
- Durability—transactions are validated, and attempts are made to confirm non-conforming ones, and transaction results are immediately detected;
- Anonymity—each user in the network is assigned a generated address (hash) that authorizes him to perform transactions;
- Audibility—each transaction relates to previous transactions, which enables verification of the processed data;
- Transparency—for public networks, transactions from any address registered in the network are available for viewing by users;
- Security—the blockchain is shared and forgery-proof;
- Invariability—it is not possible to change the data stored in the BC, moreover, each entry in the book must be confirmed by the network, each block contains the hash of the previous block, any attempt to change the data will result in rejection.

One of the key elements in favor of the use of BCT in production engineering is the security of archived and transmitted data in the network [7]. Procedures ensuring a high level of transaction security are defined by Joshi et al. [8], e.g., protection against penetration, minimum rights giving low-level access to data, risk management allows you to control risk in the network, making corrections, i.e., correcting defective codes.

The potential of using BCT in production is mainly [9]: strong cooperation (decentralization of data, equality of data management tasks, mutual trust between network participants), blockchain connection with the IOT (data autonomy), new business models (exclusion of data intermediaries, savings for operators, easier modification of production resources), smart contracts (improved efficiency in data resource management, connection with a code generator). The advantage of the technology, in terms of production, is also the cost-effective data transmission (without the need to use a central system and additional human resources) and the possibility of integrating the Internet of Things at the micro level [10]. In addition to the advantages, there are barriers to the use of BC in production [9]: inter-organizational barriers (lack of knowledge of technology, problems of cooperation in networking, shortcomings of in the competences of staff, fear of disclosing critical data), intra-organizational barriers (lack of trust, organizational limitations, lack of infrastructure), technological barriers (unclear organizational structure, complexity of network configuration, costs of BCT implementation), external barriers (BC legal uncertainty, regulatory uncertainty).

The blockchain technology is a response to the need for the above-mentioned forms of data storage and analysis, thanks to a combination of data archiving, cybersecurity, authentication, access only by authorized users, and secure and distortion-free data handling. The information flow, based on the properties of the BC (blockchain) network, facilitates enterprise resource planning and enables the integration of internal processes, especially when planning, changing the current or introducing new production. The data is collected (archived), processed, authenticated and made available to users who have an access key. Information stored in the BC network may also be treated as a database to which specific users have access. The use of the blockchain is aimed at improving company management, controlling activities, optimizing planned activities, and in the presented study, an analysis of human resources and their competences.

The objective of the paper is to present a new methodology of production scheduling and human resource management with the application of blockchain technology in order to increase the productivity of the production system.

This paper is organized as follows: a literature review on blockchain technology, production planning and human resource management is presented in Section 2. The methodology of building a blockchain network for production scheduling and human resource management is presented in Section 3. Research on scheduling production resources and managing employee competencies using blockchain technology is described in Section 4 and discussed in Section 5. Section 6 contains a brief summary of the research along with future directions.

## 2. Literature Review

Blockchain technology, initially related exclusively to the subject of the Bitcoin cryptocurrency, is gaining popularity due to the security of sensitive production data that must be carefully managed [11,12]. There are many examples of the use of devices (smartphones, smart pads) that, when connected to the network, can integrate resources into a unified internal system. The ability results from the possibility of decentralizing the structure of data registration and transmission and authentication of network users, finding applications from the Internet of Things to logistics and food technology [13]. In production management, BCT (Blockchain Technology) is useful mainly at the information-intensive stages, where production is multi-stage, contains many resources and a large number of employees at various stages of its implementation. In a production environment, blockchain can provide a trustworthy infrastructure for managing information at all stages of a product or service lifecycle.

Until now, only the integration of blockchain with the supply chain was known as part of smart contract transactions that served as a public database for product tracking [14,15]. The industry breakthrough came in 2017, when IBM and Maersk announced their collaboration to implement Blockchain Technology in the supply chain, thus integrate blockchain with IoT to transform the global supply chain [16]. Ho et al. [17] proposed a blockchain-based supported managerial platform to accurately record and track aircraft spare parts, leading to enhanced inventory control accuracy, reduced maintenance errors, and effective decision-making processes. Tian et al. [18] proposed to use blockchain technology to solve big data and privacy problems with a loose connection. Kim et al. [19] used blockchain technology to store and exchange personal health records. Feng and others [20] introduced blockchain into crowded applications serving to support life, decentralization and fault tolerance. Shrestha [21] proposed a kind of blockchain for solving critical broadcasting problems in vehicle advertising networks. There are also publications that analyze the concepts of blockchain traceability systems, typically with particular emphasis on food identification and agriculture products [22,23,24,25,26,27,28,29,30]. Blockchain technology integrated computer technologies with a decentralized structure and distributed processing [31,32,33,34]. The technology has the ability to store data in an encrypted form and transfer it between the sender and the recipient (e.g., in IPFS, InterPlanetary File System). The smart contract was proposed by Nick Szabo around 1997 and implemented using blockchain technology to create a practical application. Cai et al. [35], in their study, created a smart contract to control the availability of sensitive data in the information management system. Ji et al. [36] proposed a smart contract framework for automated scheduling for remote wind farms. The wind farm and many energy markets trust each other to transfer a certain set of real-time buy/sell volumes as agreed in the smart contract.

In the field of production management, BCT has gained interest among companies operating in the supply chain, mainly due to the ability of the integration of technology with the supply chain [37]. In the production part of the supply chain, new technologies have already been implemented, mainly to create the base and share production knowledge to increase productivity [38,39,40]. BCT can be implemented to track the life cycle of a product from row material to many years after its sale, to maximize the value of take-make-waste economy by recovering, recycling or reusing the product [41].

Various research directions related to the participation of human resources in production processes appear in the literature. The solutions for planning additional resources allow for the development of useful extensions of scheduling models and serve as a support in the selection and implementation of an appropriate tool or techniques [42]. The authors [43] discuss the use of additional, scarce resources during the operation. The authors provided a classification scheme for resource constraints and computational complexity for the configuration of parallel machines, unit tasks and the maximum completion time criterion [44,45]. The simulation of human resources allocation is presented in [46]. Authors discuss the impact of planning strategies on human resource requirements. In addition, the stages of comparing labor demand and supply in human resource planning are presented, and the most important factors limiting the use of employee resources in terms of time (work calendar) and labor law (regulations) are taken into account. General models that represent a wide range of real staff problems are presented in [47]. The work contains a comprehensive description of the data, hard and soft constraints, as well as goals. An analytical approach to personnel planning in a multi-project environment is presented in [48]. The review report published in [49] refers to almost three hundred publications dealing with problems related to staff scheduling including the characteristics of work teams, constraints, measures of performance and flexibility, as well as solution methods and areas of application. The method of project management derived from the theory of constraints as decision support tools that enable project managers to monitor and control key factors influencing, e.g., task time is described in [50]. The scope of modeling operations with multi-resource requirements, taking into account constraints of human resources is presented in [51].

According to the authors’ knowledge, this is the first paper in which the production planning and human resource management using the Blockchain Technology are considered. Fachrunnisa et al. [52] developed a blockchain-based human resource management framework to identify the skill information needed by the industry. After the skill gaps facing the sector are identified, the corporate training center makes regulations. The presented framework bridges the gaps by organizing the necessary training. In this paper, workers with necessary skills and competencies are sourced from the market and resources of a virtual enterprise. The presented framework of human resource management meets the needs of flexible and agile management systems operating in industry 4.0. The remaining literature [53,54] outlines the need to develop a human resource management system and proposes a framework for the use of blockchain.

Compared to the analyzed literature on human resources management and production scheduling, the distinct nature of the work presented in this paper consists of:

- A new methodology of production scheduling and human resource management with the application of blockchain technology for virtual enterprises;
- A resource (an employee) evaluation method based on the resource work history and determination of its current value within individual competencies;
- The application of open source blockchain to search for a suitably qualified employee, which allows for better use of the existing workforce and improvement of the quality of manufactured products in VE.

## 3. Methodology of Building a Blockchain Network

**Figure 1:**
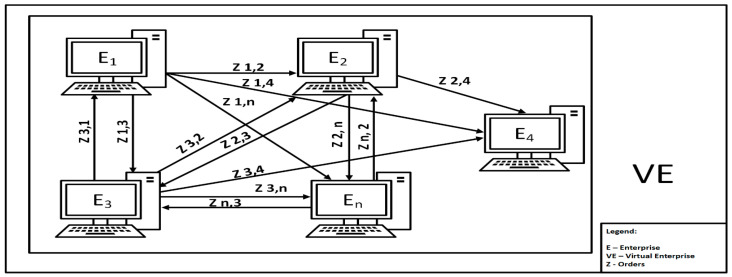
Open tender for the execution of a production order using consortium blockchain.

**Figure 2:**
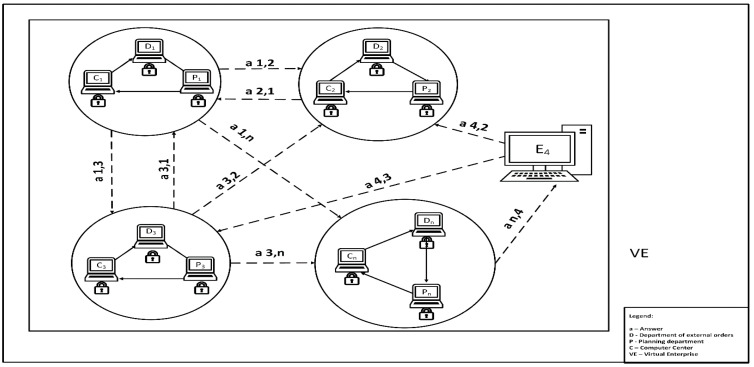
Responses to the tender with preliminary terms of the contract using consortium blockchain.

Based on the blockchain classification presented by Lin et al. in [55], we propose the application of consortium blockchain for the production planning and human resource management network. Consortium blockchain contains a pool of selected nodes that allow data to be added to the chain, and their reading can be open or private:

- An open tender for the execution of a production order using consortium blockchain with open reading is presented in Figure 1. Each enterprise has an equal right to generate a block for the execution of a production order (tender for employee qualification) available to other enterprises in the Virtual Enterprise. Orders are available to anyone interested. Tender notices contain encoded information such as: what is to do, when, in what quantity, what are the technological requirements, and what are the required human competencies. Let us analyze order z_1_ generated by enterprise E_1_. Enterprises read the book with order z_1_: order z_1,2_ was read by enterprise E_2_, and order z_1,n_ was read by enterprise E_n_.
- Responses to the tender with preliminary terms of the contract using consortium blockchain are presented in Figure 2. In response to each contract, the following information is included: the identity of the available employee, availability time, valuation of historical competences held by the resource (in qualitative and quantitative terms) recorded along with their valuation, and a working time calendar.

Let us analyze order z_1,2_ generated by enterprise E_1_ and answer a_2,1_ generated by enterprise E_2_. After reading the order z_1,2_ by Computer Center C_2_ of enterprise E_2_, the next block is generated for private reading by the Orders Department D_2_ of enterprise E_2_. The Orders Department D_2_ checks the contracts for the correctness of the data and generate the block for private verification by the Planning Department P_2_. The Planning Department P_2_ performs an assessment of compliance of production tasks with the capacity (if the is executed in the parent company) and availability of employees with the required competences and/or availability of other resources (machines). After generating block with the feasibility approval, the Computer Center C_2_ generate the block with response a_2,1_ for order z_1,2_. The enterprise does not respond if it does not have resources available in the required time.

**Figure 3:**
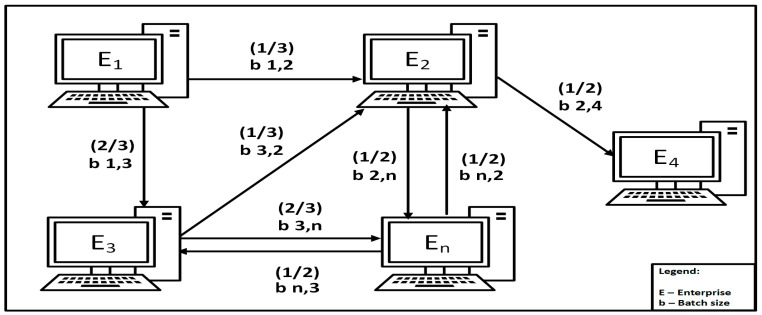
Generating orders to companies in the VE according to the highest competence rule using consortium blockchain.

Finally, blocks with full or partial batch size contracted production orders are generated with private reading for companies in VE. Computer centers generate orders with private reading for companies according to the principle of the highest competence presented in the next chapter. The Computer Center C_1_ generates the block for order a_1,3_ with batch size b = 2/3 for privet reading by enterprise E_3_ and order a_1,2_ with batch size b = 1/3 for privet reading by enterprise E_2_ (Figure 3).

**Figure 4:**
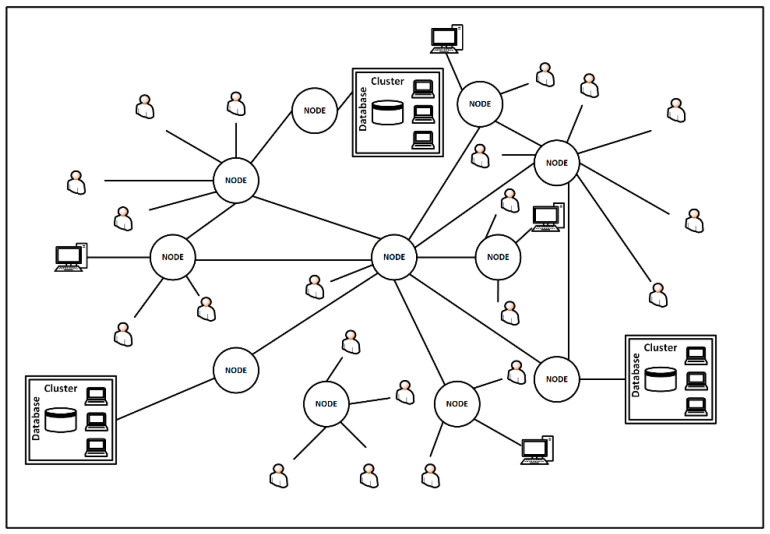
Decentralized blockchain network in the VE.

A BC network is often referred to a distributed network (Figure 4). Such a network consists mainly of nodes, i.e., individual systems (of computer centers of enterprises) that store and care for the validity of transactions, and users, i.e., entities or people who have the ability to read the book.

A chain is created when a node holding a block connects it to a new block. Additionally:

- A database is created, which is entered into the system by persons designated for this purpose of computer centers of enterprises;
- Information about the newly created block is sent to each node in the network;
- All network nodes must verify and then approve the transaction in accordance with the agreed access key;
- After validation, the block is added to the chain. This step completes the operation and ensures its permanent record.

**Figure 5:**
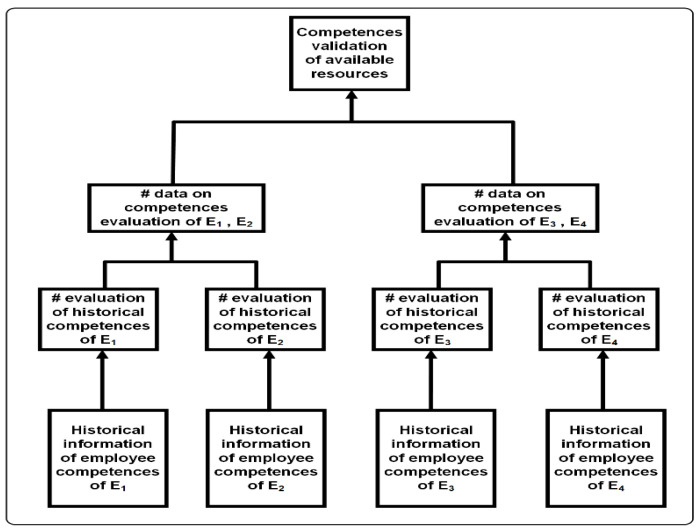
Merkle tree for employee competency assessment.

In BC, each standard block contains data such as: block number, hash of the current block (generated from the data contained in the block, most often using the Merkle tree (Figure 5), hash of the previous block, timestamp, transaction list. The competencies of employees of enterprise E_1_, E_2_, E_3_ and E_4_ are validated on the basis of historical qualitative and quantitative data of the employee’s competences in parallel. Only available employees are included in the assessment.

**Figure 6:**
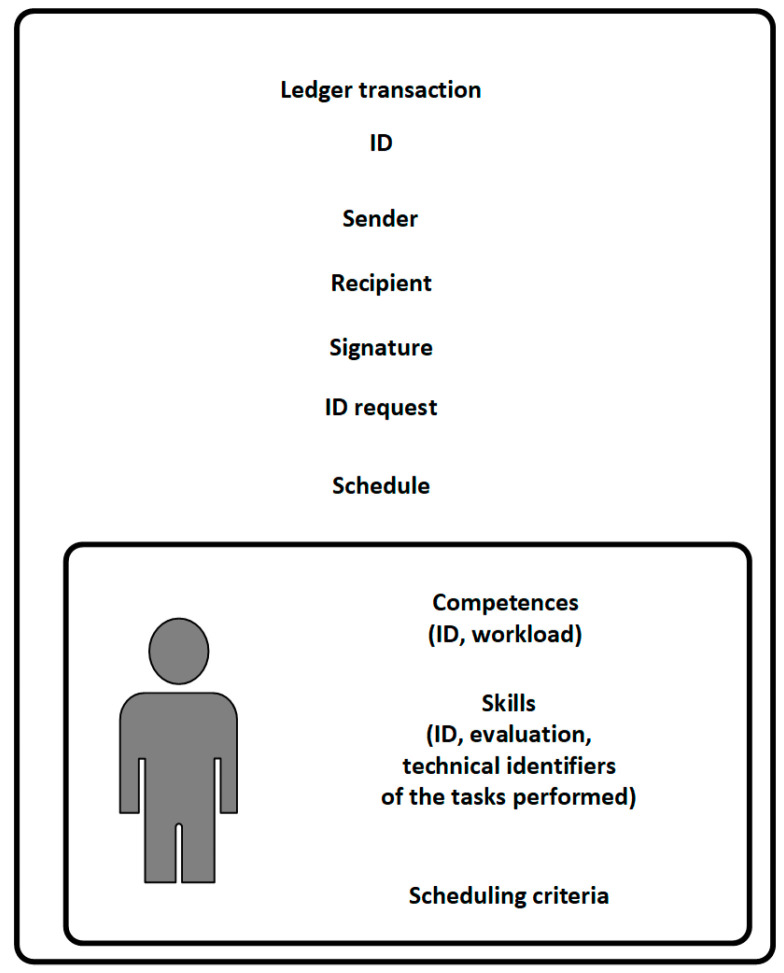
The content of the employee competency assessment in response to an open tender.

A fully prepared assessment of competencies (Figure 6) includes such information as:

- ID—the assessment identifier generated on the basis of data contained in it,
- Sender—public key of the node preparing the open tender (demand for an employee),
- Recipient—public key of the manager of the planning department creating the assessment,
- Signature—a digital signature made by a node (the manager of the planning department),
- Request ID—identifier of the request sent by the manager preparing the open tender,
- Information about the added availability time (schedule) and evaluation of employee including competences and technical skills of the employee (machine operation, the ability to read technical drawings, ongoing projects, the level of difficulty of the tasks performed, the level of involvement and position in the project, courses held),
- Criteria for selecting the given data.

**Figure 7:**
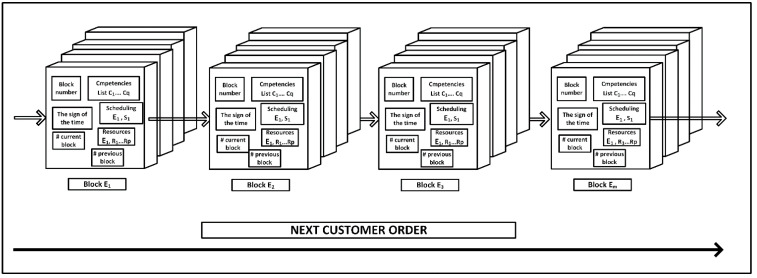
Blockchain for reacting to subsequent orders for employees and building consensus. # marks the beginning of a comment that continues to the end of the physical line. # precedes the reference to the previous and the next block so that the new data that is applied will look up the order of the data and can bind them together in a blockchain.

Each new book begins with a block named Genesis Block, which specifies the beginning and imposes the necessity to add additional blocks to the chain. An example blockchain for four employees selection by consensus to execute four subsequent orders (open tenders) is shown in Figure 7. In each book, the procedure for responding to a new order for an employee and building consensus is repeated.

**Figure 8:**
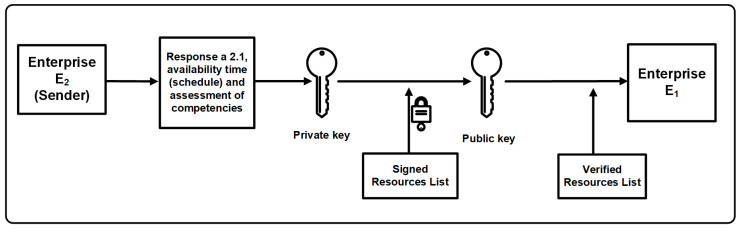
The procedure of signing and verifying signatures for the question about employee availability.

The blockchain operation consists of a few simple steps:

1. The sending node prepares new data as an order for employee availability and broadcasts it in the network.
2. The receiving node verifies the transaction (question about employee availability or answer to the question) and the data contained therein. Each transaction must be signed and authorized using asymmetric cryptography [56]. The private key is used to sign the transaction, while the public key is used to identify the user (user’s address) and verify the signature generated with the use of the private key, which allows you to control whether the user who sent the message is its author. When the transaction and the entered data passes the validation process, the node responds to the sending node, which results in the transaction being saved in the block. The procedure of signing and verifying signatures in answer a_2.1_ along with the assessment of the availability and competences of employees is presented in Figure 8.
3. Recording of the appropriate number of validated transactions in the local block causes the nodes to start the block confirmation procedure (in accordance with the terms of the consensus adopted in the network).
4. Completion of the block confirmation procedure with the appropriate number of nodes and executing the consensus algorithm based on the priority rules (presented in the next section) saves the block to the chain.
5. Each node in the BC network writes locally the committed block and includes it in its chain.

## 4. Methodology of Building Consensus

**Figure 9:**
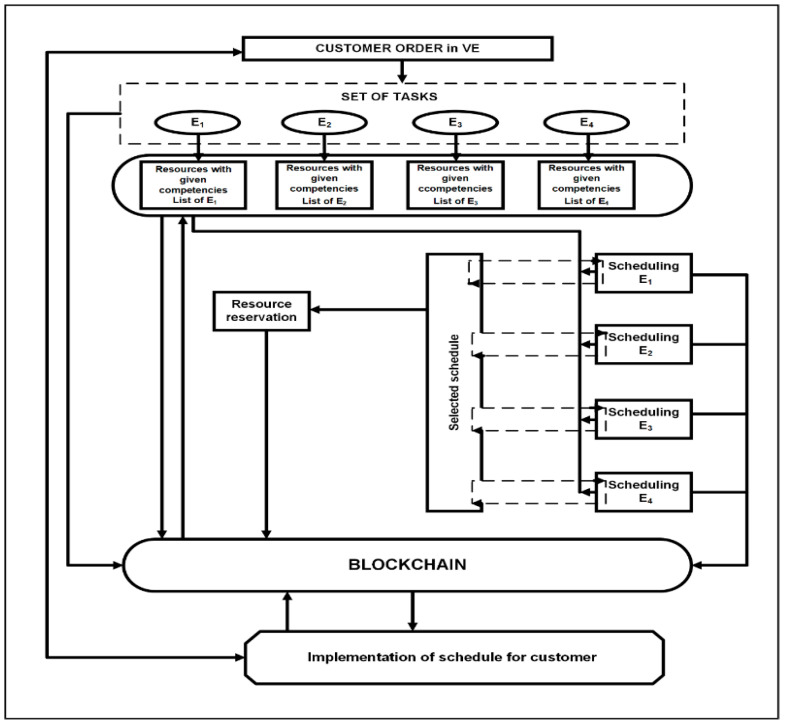
Conceptual model of production and human resources scheduling.

Nodes placed on a blockchain (BC) network communicate with each other and define a consensus in terms of a properly made schedule. Any node in the network can generate the partial schedule for available employee and machines. The first node initiates the transaction. Schedule is added to the blockchain (saved) when most of the nodes in the network confirm the correctness of the presented schedule generated from the matched partial schedules. Such action is tantamount to its approval. The action is performed in the cloud or sent directly to the user, provided that it only contains tasks information from a given user. In the block network, each node generates its own schedule and compares it with the schedule proposed by its predecessor in order to align the dates of the predecessors and successors. The conceptual model of blockchain scheduling including employee competencies is presented in Figure 9.

Companies carry out tasks independently or as part of consortia or virtual enterprises. Each employer, associated in a cluster or union, has access to the ledger, provides data on the organization’s own resources (employee skills and competences) from its own database. Each employer provides data on projects or processes to a selected participant in the network (e.g., another company looking for employees with specific competencies or knowledge in a given field) in order to strengthen the reputation of the organization, and thus partially define the trust between the parties. An enterprise or a group of enterprises builds a production schedule based on a given project or a list (package) of production orders, appropriate requirements for production resources (workstation, machines and other means of production, tools and equipment as well as human resources with given competences).

**Figure 10:**
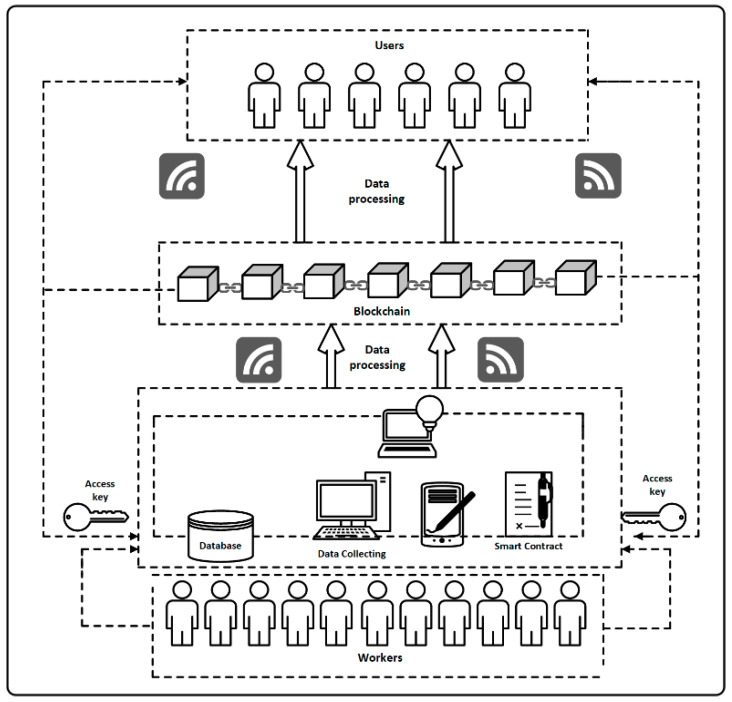
The process of data circulation containing information about employee competencies in blockchain technology.

This selection is not accidental, it requires optimization measures (from the point of view of both time and costs, properly selected resources are of key importance). These resources are at the disposal of one enterprise, group of companies on an outsourcing basis or in a pool available under leasing (employment agency). Therefore, for effective database management, a common register of competences and resource availability is needed. The traditional workflow requires contractors to ask about their current status each time. Blockchain-based database implementation consists in entering the records of interest to us, then the database is searched in relation to the information contained in it, and then the results are presented along with annotation about the available data (Figure 10).

The process of collecting data on employee competencies in the block network is presented in Figure 10. The information that is entered and then shared with users regarding human resources is: what is the scope of the employee’s duties, what machines does he work on, what skills does he have, what is his professional experience in the field of production, what courses does the employee have, what projects he manages and what functions does he perform within projects, what experience does he have in international and inter-departmental projects, etc. Information on the competences and skills of employees (collected on devices such as: computers, tablets) is accepted by users by means of a smart contract and transferred to a collective database. Then, the data is processed and saved in a blockchain, to which selected users have access. The users can view, book and rent the resources they need.

**Figure 11:**
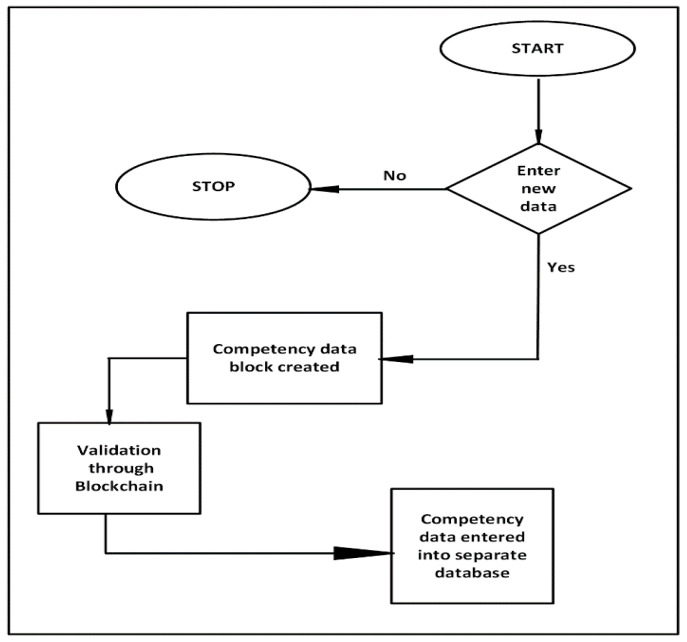
The process of collecting data on employee competencies in the block network.

The stages of the process of entering and functioning of data in the network are shown in Figure 11. In the first stage, a query is generated that initiates the process of introducing new data about the employee, his competences, skills and availability. If no new data is available, the process is terminated. If a user (e.g., a company) decides to introduce new data to the network, a data block with employee competencies is created. Subsequently, the data is validated and entered into a designated dedicated database. With each employee, a set of his skills is created in the central database of competences (e.g., machine A, machine B, Machine C, qualifications X, course Z) and in the database with the “busy/free” status.

**Figure 12:**
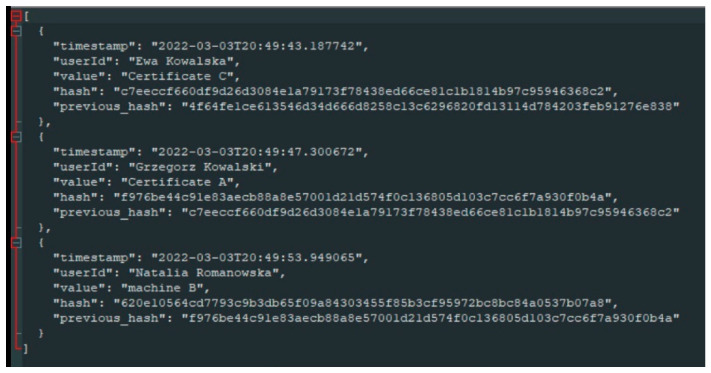
A piece of encoded data in BC.

BC example with the encoded data on employee competences is presented in Figure 12. The presented BC is coded using the Python programming language. Line “timestamp” means the date and time of entering specific competency data. Line “User Id” describes employee’s name (first and last name). Line “Value” codes the value of the assessment carried out for the employee, taking into account his competences and skills. Line “Hash” describes the identifier of entered data files. Line “Previous hash” contains the previous identifier of the entered data files. This field is located inside the block header and thus affects the hash of the current block, ensuring security against unauthorized breach of the network. Hashing randomly generated numbers to obtain a specific hash value containing a series of leading zeros makes it difficult for unauthorized people to hijack the network to obtain information about the data contained in it.

The use of a shared data registered in BC technology enables:

- Quick access to the necessary data to optimize the selection of production resources,
- Automation of the process of selecting resources for tasks,
- The possibility of a reliable assessment of human resources competences—each “employment” of a resource is associated with a feedback evaluation of its work (multi-criteria assessment: correctness of the task performance, commitment, punctuality, hard and soft competences),
- Guarantee of resource reservation (transactions),
- Compliance with data security and confidentiality procedures,
- Lower costs of IT infrastructure,
- Freedom of access to human resources,
- The possibility of task-based employment of qualified staff,
- Easy development of the structure of related enterprises and employment agencies as part of joint production projects.

The proposed solutions support decision-making in the field of production planning and scheduling, and improve the process of answering standard questions:

- What are the production capabilities for the given resource constraints?
- What subset of resources is sufficient (or the most beneficial) to implement the adopted production plan?
- How to select the production range in order to maximize the use of resources?
- Is it possible to execute a new order in a specified quantity and time?

Accepting a specific solution starts a new block related to the reservation of resources for the tasks provided in the schedule. A block is created after completing the schedule, task or order. In the register, another entry—a record updates the employee’s competencies. The proposed solution uses the BC register to store data on the availability of employees, reserve them for specific tasks and accumulate their achievements, competencies of various dimensions.

Every time an entry is refreshed, similar changes are reflected in all other ledgers, thus preserving the decentralized, simple and communicated nature of blockchains. To ensure traceability using blockchain technology, a link must be established between the blockchain platform and the physical product. This approach assumes that all data refer to the resource, to the employee’s competences.

Smart contracts generate unique identification codes. The numbers of the entered data must be clearly assigned to the owner and be able to change the owner in the event of dismissal or transfer of the employee to another entity associated in the cluster of enterprises. The entity that is the “owner” the process of creating a competency unit should have access to a smart contract in order to create a virtual identity for a given employee. Since blockchain can record data chronologically, the records of previous owners will prevent the use of unverified information. It also provides proof of authentication in the event of conflicts. Each stakeholder involved in the employee competence chain has an account to have free access to the network. Data generated by employees on the competences of the crew can be sent to a smart contract for verification. In the aspect of information transparency, the generated data can be transmitted on blockchain, allowing authorized stakeholders to read and verify it on their own. A typical smart contract consists of three components: contracts and logic in the blockchain, the user interface, and back-end resources such as off-blockchain storage [57].

**Figure 13:**
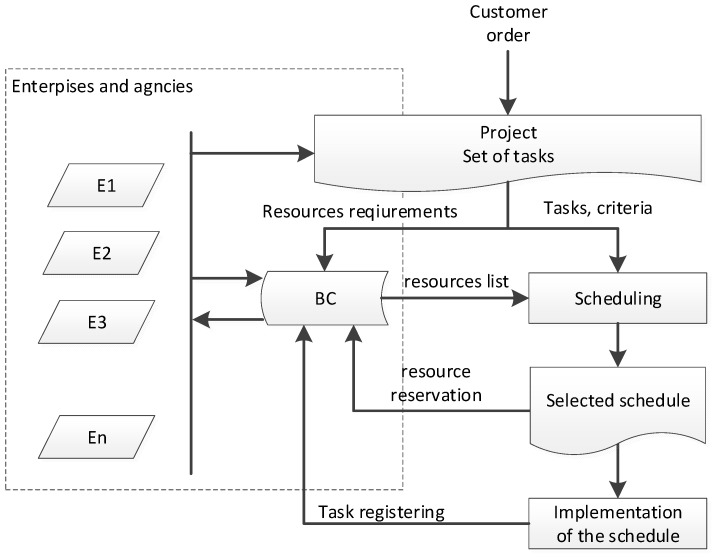
Production scheduling process with the use of BC technology to manage employee competencies.

The assumed level of task complexity hinders the process of manual selection of resources and prevents the optimization process. Therefore, advanced task planning algorithms are needed along with mechanisms that enable these algorithms to be supplied with appropriate, up-to-date data. The production scheduling process with the use of BC technology to manage employee competencies is summarized in Figure 13.

Accepting an order for scheduling requires designating a set of available resources that can be used for its execution. For this purpose, an inquiry is made to the BC registers of all partners participating in the blockchain network. Resources with the required, specific competencies are searched. The main assumptions are:

- An operation may require more than one resource to be reserved, e.g., groups of employees, several machines,
- The operation may require the reservation of resources with different competencies (machine + tools + workers with different competencies).

Each shared resource should have its Genesis Block (GB) initialized. In terms of information stored in the GB block, required for planning activities, the competences held by the resource (in qualitative and quantitative terms) are recorded along with their valuation and the working time calendar. The individual periods (Ok_i_) are described by the following:

Ok_i_ = (tb_i_, te_i_, C_k_, v_i_) (1)

where:

i—the index of the work period,
tb_i_, te_i_,—start and end time of the work period, respectively,
C_k_—k-th competence within which the resource worked,
v_i_—generalized value of the resource’s work evaluation, v_i,k_ ∈ <0;1>.

The generated resource list is used for scheduling. Depending on the adopted procedure, there are two possible scenarios for assigning resources to tasks: monolithic or hierarchical. In the monolithic approach, the activities of allocating operations to resources and scheduling are carried out jointly. Additionally, the selection of the resource occurs simultaneously with the determination of the start time of the operation. In the hierarchical approach, all operations are first allocated to resources and then the start times are calculated in the appropriate order. The selection of resources is therefore based on their value, the need for an even load (hierarchical, monolithic approach) and/or availability (monolithic approach). Since the BC contains all the historical activities of a given resource, it is possible to use it to determine the current value of the resource within each of the competencies required.

The registration of the above data enables a later review of the resource’s work history and determination of its current value within individual competences. The resource valuation method is:

(2) $$v_{z,k}^{\mathrm{tn}}=\overset{T_{2}}{\underset{T_{1;\;\mathrm{ik}=k}}{\sum }}\left(\mu \left({\mathrm{te}}_{i,k}\right)\left({\alpha v}_{i,k}+\beta \frac{\left(({\mathrm{te}}_{i,k}+1)-{\mathrm{tb}}_{i,k}\right)}{\sum_{T_{1;\;i}}^{T_{2}}\left(({\mathrm{te}}_{i}+1)-{\mathrm{tb}}_{i}\right)}\right)\right),$$

where:

i, n, x—time,
$v_{z,k}^{\mathrm{tn}}$—assessment of professional experience of resource z in terms of competence k, in time t_n_,
$v_{i,k}$—evaluation of the i-th period of work with competence k,
$t_{n}$—the moment of determining the grade,
tb_i,k_, te_i,k_—start and end time of the work period with competence k, respectively,
T_1_, T_2_—beginning and end of the considered period of historical data analysis,
$\mu \left(t_{x}\right)$—coefficient of the work period value at time $t_{x}={\mathrm{te}}_{i,k}$,
A—importance coefficient of the value of competency k, α ∈ <0;1>,
B—importance coefficient of duration time of work, β ∈ <0;1>.

According to the above Formula (2), the evaluation of the value of employee competencies at time t_n_ is based on historical data within a fixed range <$T_{1},{\;T}_{1}$>, considering the time that has elapsed since the activities performed within competence k, using µ(t_x_). This allows for the differentiation of partial assessments and a preference for the value of competencies that have been acquired recently, which is particularly important if a given employee improves the quality of his work over time. An example of calculating the value of a single competency of an employee is presented in Table 1.

In this example, the register period was assumed to 12 time units, the values of the µ(t_x_) coefficient in the range (0.3–1), decreasing linearly with time (older periods have a lower coefficient). Coefficient of the work period value µ(t_x_) at end time t_x_ = te_ik_ of competence k is calculated. Two activities related to competency k = C_1_ were registered and assessed in the employee’s schedule: Ok_1_ = (1,4, C_1_, 0.7) and Ok_2_ = (9,10, C_1_, 0.9). The values of 0.7 and 0.9 were reached during the evaluation after each activity, respectively. The final assessment of competency C_1_ is 1.439 for the assumed values of α = 1 and β = 1.

The value of a resource in terms of competences is obtained from periods representing work history (BC). Resource must be available to engage in a task. If there are more resources available than the requirements of the planned task, a greater number of variants may arise at the scheduling stage. The created schedules require evaluation and selection of a solution for implementation.

The selection of the schedule variant for implementation requires registration in the BC network due to the necessity to reserve terms for selected resources. Then, upon completion of the scheduled tasks, the resources BC are updated with a new period (1) with a current assessment of their competences. The process of resource selection is repeated for another task.

## 5. Numerical Example and Discussion

**Figure 14:**
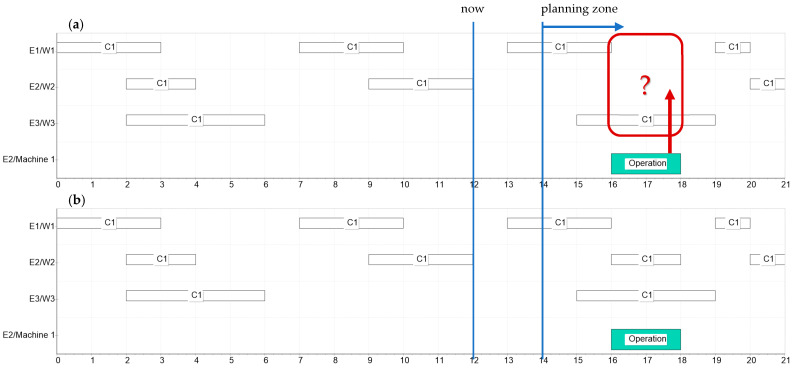
The scheduling of the additional human resource for an operation based on work experience within competence C_1_, (**a**) two resources are available: worker 1 of enterprise 1 and worker 2 of enterprise 2, (**b**) worker 1 is selected to execute the operation according to Formula (2).

Figure 14 shows an example of reserving an additional human resource for an operation. The presented schedule lists the work history of resources within competences C_1_ (from 0 to 12 time unit) and the planned workload in the near future (reservations from 14 to 21). At enterprise E_2_, an operation is planned on Machine 1 for period of 16–18, which requires the participation of an operator with C_1_ competence. For the selection of the resource, the Formula (2) was used, taking into account the records of previously performed tasks within C_1_ competencies, by each resource, up to 12 time units (“now”).

Table 2 presents data based on information from the BC: schedule and evaluation value of individual tasks. The values T_1_ = 0, T_2_ = 12 (now), α = 1, β = 1 were adopted for the calculations, which means a full history and the equivalent: seniority and job evaluation. Different parameter values can be used each time when selecting resources for different operations.

Based on the determined value, the employee W2 of enterprise E2 was selected and the date was booked in accordance with the date of the operation (Figure 14b).

The presented methodology of production scheduling, taking into account the competences of employees using Blockchain technology, allows for faster and more effective planning thanks to the elimination of all intermediary channels in the flow of transaction. It is also possible to perform balancing tasks in parallel with production capacity in entities belonging to the virtual enterprise. Optimization is based on multi-resource production data with faster verification of available resources and their competences. The remaining comparison of the information flow on production tasks and employee competencies in the traditional and blockchain systems is presented in Table 3.

Blockchain technology does not have many applications in enterprises due to certain challenges such as scalability, high energy and computing power, throughput, latency, high setup cost and lack of standardization [58]. The disadvantage is that the consensus protocols used to maintain blockchain integrity have to be executed every time to add new blocks. As new blocks are constantly added to the chain, it grows, which means that more energy and enormous computing power are needed [41]. In the presented approach, the local consensus is concluded by the enterprise sending the open tender, taking into account the available dates or resources of the corresponding enterprises. Another problem concerns the time it takes to generate the next block of transactions in the chin, and the current delay is around 10 min, which is much longer than in traditional systems [59]. Moreover, each node in the blockchain basically performs the same task and due to the lack of sharing the executed task cannot be parallel. Within the global schedule, common consensus causes no sharing, which makes it impossible to perform parallel tasks. In the proposed approach, each node in the blockchain performs the same task, i.e., responds to subsequent orders by searching for available employees (Figure 7). Local schedules are built in parallel, which does not require the involvement of all companies each time.

Researchers believe that the arising version of blockchain 4.0 is to ensure interoperability and is dedicated to enterprises due to the ability to overcome challenges such as scalability, adaptability and affordability.

## 6. Conclusions

In the paper, the methodology of production scheduling and human resources management with the use of BC technology was presented. The presented approach differs significantly from the existing commercial Internet solutions and is interesting for various sectors of the economy, including the production management sector.

Thanks to the automation of the process of selecting human resources for production tasks with the use of BCT, all channels intermediating in the flow of production transaction have been eliminated. The presented methodology of scheduling production with the use of blockchain technology allowed for faster and more effective planning of human resources. Reliable data on employee competencies was collected and assessed. The optimization process was based on multi-resource production data with faster verification of the available resources and providing information about deficiencies. The aim of the paper was also to increase the transparency and the availability of employees in the cooperating production networks.

In the presented approach to the assessment of the productivity, the criterion of the maximum use of existing resources (machines and employees) of the enterprise in BC network was applied. Time criteria such as makespan and tardiness are also needed for consideration. Therefore, in the future, multi-criteria optimization methods will be developed to build consensus in the problems of production and human resources planning. Additionally, different online planning algorithms will be developed and compared to achieve consortium members’ consensus on production and human resources planning.

## Figures and Tables

**Table 1:** An example of calculating the value of a single competency of an employee using Equation (2).

Period t_x_	1	2	3	4	5	6	7	8	9	10	11	12	Sum
µ(t_x_)	0.300	0.364	0.427	0.491	0.555	0.618	0.682	0.745	0.809	0.873	0.936	1.000	
competence	C_1_	C_1_	C_1_	C_1_					C_1_	C_1_			
(te_i_ + 1) − tb_i_				4						2			6.0
v_i,k_				0.7						0.9			
Partial eval.				0.507						0.931			1.439

**Table 2:** An example of calculating the value of a single competency of employee W of enterprise E.

Resource	Period t_x_	1	2	3	4	5	6	7	8	9	10	11	12	Sum
	µ(t_x_)	0.300	0.364	0.427	0.491	0.555	0.618	0.682	0.745	0.809	0.873	0.936	1.000	
E1/W1	(te_i_ + 1) − tb_i_			3							3			6.0
E2/W2	(te_i_ + 1) − tb_i_				2								3	5.0
E3/W3	(te_i_ + 1) − tb_i_						4							4.0
E1/W1	v_i,k_			0.8							0.9			
E2/W2	v_i,k_				0.7								0.8	
E3/W3	v_i,k_						1.0							
E1/W1	Partial eval.			0.448							1.004			1.452
E2/W2	Partial eval.				0.426								1.050	1.476
E3/W3	Partial eval.						0.824							0.824

**Table 3:** Comparison of the flow of information on production tasks and employee competencies in the traditional and blockchain systems.

**Stage**	Traditional	Blockchain
Resource requirements	1. inquiry for own resources 2. verification and assessment of own resources 3. request for external resources (3–6 if needed) 4. waiting for an answer 5. verification and assessment of the use of external resources 6. assessment of profitability and costs of using internal and external resources. 7. final selection of resources	1. parallel query for internal and external resources 2. simultaneous evaluation of profitability and costs of using internal and external resources. 3. final selection of resources
Resource reservation Task registering	own registers (the reserved terms of the resource are visible within the entity) external registers is visible in two entities (contracting party—contractor)	shared registers (reserved resource terms visible in the entire group)
Competences and their assessment	available in the parent entity	available to all authorized entities
History of competences	possible manipulations	Reliable
Personal data protection	Limited	full protection
Database	centralized database is vulnerable to cyber attacks	decentralized database is immune to cyber attacks

## Data Availability

Not applicable.
